# Luminescent Metal–Organic Frameworks for Electrochemiluminescent Detection of Water Pollutants

**DOI:** 10.3390/ma16237502

**Published:** 2023-12-04

**Authors:** Milica Sentic, Ivana Trajkovic, Dragan Manojlovic, Dalibor Stankovic, Maria Vesna Nikolic, Neso Sojic, Jasmina Vidic

**Affiliations:** 1 Institute of Chemistry, Technology and Metallurgy, National Institute of the Republic of Serbia, University of Belgrade, Njegoševa 12, 11001 Belgrade, Serbia; milica.sentic@ihtm.bg.ac.rs (M.S.); trajkovic@ihtm.bg.ac.rs (I.T.); 2 Faculty of Chemistry, University of Belgrade, Studentski trg 12-16, 11000 Belgrade, Serbia; manojlo@chem.bg.ac.rs (D.M.); dalibors@chem.bg.ac.rs (D.S.); 3 Institute for Multidisciplinary Research, University of Belgrade, Kneza Viseslava 1, 11030 Belgrade, Serbia; mariavesna@imsi.bg.ac.rs; 4 Bordeaux INP, ISM, UMR CNRS 5255, University of Bordeaux, 33607 Pessac, France; neso.sojic@u-bordeaux.fr; 5 INRAE, AgroParisTech, Micalis Institute, UMR 1319, Université Paris-Saclay, 78350 Jouy-en-Josas, France

**Keywords:** metal organic frameworks, luminescence, electroluminescent detection, water pollutants

## Abstract

The modern lifestyle has increased our utilization of pollutants such as heavy metals, aromatic compounds, and contaminants that are of rising concern, involving pharmaceutical and personal products and other materials that may have an important environmental impact. In particular, the ultimate results of the intense use of highly stable materials, such as heavy metals and chemical restudies, are that they turn into waste materials, which, when discharged, accumulate in environmental water bodies. In this context, the present review presents the application of metal–organic frameworks (MOFs) in electrochemiluminescent (ECL) sensing for water pollutant detection. MOF composites applied as innovative luminophore or luminophore carriers, materials for electrode modification, and the enhancement of co-reaction in ECL sensors have enabled the sensitive monitoring of some of the most common contaminants of emerging concern such as heavy metals, volatile organic compounds, pharmaceuticals, industrial chemicals, and cyanotoxins. Moreover, we provide future trends and prospects associated with ECL MOF composites for environmental sensing.

## 1. Introduction

Pollution from different sources due to extensive urbanization and industrialization is placing the global water budget under pressure by reducing the water available for beneficial use [1]. The demand for water has increased tremendously in agricultural, industrial, and domestic domains, resulting in the huge impact of natural and anthropogenic substances that are constantly released into the environment. Prime agricultural land is being lost to urbanization, threatening the aquatic and terrestrial ecosystems due to the increased use of chemicals. Anthropogenic sources of pollution are growing in number in line with progressing human development. Industrial, agricultural (pesticides and fertilizers), and mining activities, accompanied by construction, fuel and coal burning, traffic emissions (i.e., exhaust gases), and sewage waste, are the most customary pollution sources.

Amid different pollutants, even low concentrations of heavy metals have raised health concerns owing to their capability for hazardous bioaccumulation through food chains via the formation of metal–organic complexes [2]. The greatest number of non-degradable potentially toxic elements (PTEs) include Arsenic (As), Cadmium (Cd), Chromium (Cr), Copper (Cu), Mercury (Hg), Nickel (Ni), Lead (Pb), and Zinc (Zn), pose a serious threat to the ecosystem [3] when they are above the maximum allowable limits and are listed as priority pollutants requiring control by the U.S. Environmental Protection Agency and the EU Commission. These elements are found in varying amounts throughout the Earth’s crust as naturally occurring components in the water–sediment environment. Their geochemical levels in sediments are, thus, expected to be relatively low [4]. However, when heavy metals are discharged into aquatic environments from anthropogenic sources, they accumulate between the aqueous phase and the sediments. 

Volatile organic compounds (VOCs) are persistent and important organic pollutants that can lead to groundwater contamination. VOCs include chlorinated solvents (e.g., hydrogen sulfide, trichloroethene, carbon tetrachloride) and petroleum hydrocarbons, especially BTEX (benzene, toluene, ethyl-benzene, and mixtures of o-, m- and p-xylene) compounds. These volatile aromatic compounds are severely toxic to aquatic organisms if contact is maintained. They are generated by the incomplete combustion of organic matter, which is often found in discharges and petroleum products (vehicle exhaust, coal burning and residential heating, waste incineration, petroleum refining processes, and aluminum production). Groundwater contaminated with VOCs can potentially affect freshwater aquatic ecosystems as it can discharge for a long period of time to surface water bodies. Gasoline can contain large amounts of BTEX (up to 40%), and therefore, BTEXs are used as indicators of the gasoline contamination of sediment samples when gasoline contamination is suspected. 

Aquatic ecosystems are also under threat from the bioaccumulation of other sustainable freshwater contaminants, which are classified as contaminants of emerging concern (CECs) [5,6,7]. Under this broad family of chemical pollutants, we find synthetic chemicals that could have an impact on human health or ecology. Endocrine-disrupting chemicals or endocrine disruptors (EDCs) and non-steroidal anti-inflammatory drugs (NSAIDs) represent two main CEC subgroups. These pollutants can originate from agriculture, urban runoff, or ordinary household products (disinfectants, fragrances, pesticides) and pharmaceuticals dispatched to sewage treatment plants and then discharged. One main concern with CECs is that existing traditional wastewater treatment processes are ineffective in their removal. The most common include hormones endocrine-disrupting chemicals (estrone, 17ß-estradiol, 17α-ethynylestradiol, testosterone), disinfection by-products, fluorinated substances (bisphenols, phthalates, synthetic estrogens), pesticides (glyphosate and organophosphorus pesticides), and antibiotics. 

Finally, waterborne pathogenic bacteria and the mass occurrence of cyanobacteria blooms due to anthropogenic activities in freshwaters, including drinking water reservoirs, threaten human health and the environment because of their toxin-producing qualities [8]. The most frequently occurring and studied biologically active cyanobacteria toxins are anatoxins (ATXs) and microcystins (MCs), whose toxicity is a major cause of concern in the scientific community and the World Health Organization [9,10]. Anatoxin-a(S) represents an extremely potent natural neurotoxin generated by freshwater cyanobacteria, while exposure to microcystins, which are chemically very stable, leads to liver dysfunction, hemorrhage, and, in acute doses, causes cancer. Due to chronic low-dose exposure, microcystins are cancer promoters. The stability of MCs is attributed to their cyclic structure, which remains unchanged after a few hours in boiling water and even for several years at room temperature if they are in a dry state. Indeed, microcystins are not readily removed from drinking water via conventional treatment methods, which indicates the importance of toxin detection and monitoring in freshwater [11,12].

With such a vast variety of pollutants, the control of water–sediment environments remains a priority and a problem, which has been highlighted in a considerable number of scientific publications [13]. While plans to minimize global environmental pollution exist, the contamination of water and its sediments is stressing the urgency of technological advances in materials for pollutant-sensitive detection and their elimination. New sensing materials and methods showing outstanding performance, reflected by high sensitivity and selectivity, rapid detection, and ease of use, in comparison with traditional expensive chromatography with complex pretreatment and long test times, are decidedly needed. A wide range of micro- and nanomaterials, including nanocarbon materials (carbon nanotube and graphene), metals and metal oxides, semiconducting materials, quantum dots, and polymers with different characteristics, have been applied in environmental monitoring sensors [14,15,16,17]. Among these advanced novel materials, metal–organic frameworks (MOFs), also recognized as porous coordination polymers (PCPs), have attracted intense attention due to their excellent physicochemical characteristics owing to the coexistence of crystallinity and porosity [16,18,19,20]. The rational design of MOFs could especially provide innovative emitters or luminophore carriers for the hybrid analytical method of electrochemiluminescence (ECL). ECL combines light-emission detection with orthogonal electrochemical initiation [21,22,23]. The classical and most exploited ECL system is composed of tris(bipyridine)ruthenium (Ru(bpy)_3_^2+^) as the luminophore and tri-*n*-propylamine (TPrA) as the sacrificial co-reactant [24,25,26,27,28,29,30]. This ECL system can be significantly improved by utilizing nanomaterials [31,32,33,34,35,36,37,38,39,40,41,42]. Accordingly, ECL-based MOFs provide a new prospect for highly sensitive and targeted bioanalysis combined with functional nanomaterial design and controllable and tunable photophysical and photochemical properties through the structural modifications of organic linkers, metal clusters, and guest species [43]. Among other benefits, ECL MOFs enable the re-use of potassium persulfate as a non-toxic co-reactant in comparison to TPrA while providing the same or better sensitivity, thus making the whole system more environmentally friendly. In addition, the nanoconfinement that occurs in such mesoporous materials based on the intensity of ECL has been imaged and spatially resolved with a remarkable spatial resolution. Liu’s group showed that the ECL signals were very stable even in biological media, allowing single biomolecule imaging [43]. The high sensitivity of ECL sensors based on MOF luminophores allows the efficient detection of water pollutants, which are typically present in low amounts in water bodies. This set off the recent development of novel MOF materials for ECL and new applications, especially sensing and imaging [35,39,44,45,46,47,48,49,50]. 

In this review, we present recent developments in the development of luminescent MOF-based ECL sensors for water pollutant detection. First, we provide a brief description of the ECL method. Second, we introduce different MOFs as carriers of ELC emitters, including luminol, Ru(bpy)_3_^2+^, and their derivatives. Third, we outline promising applications of MOF-based ECL in water monitoring. Finally, we discuss some perspectives on the synthesis and applications of MOF luminophores for ECL sensors. 

## 2. ECL Sensors

**Figure 1:**
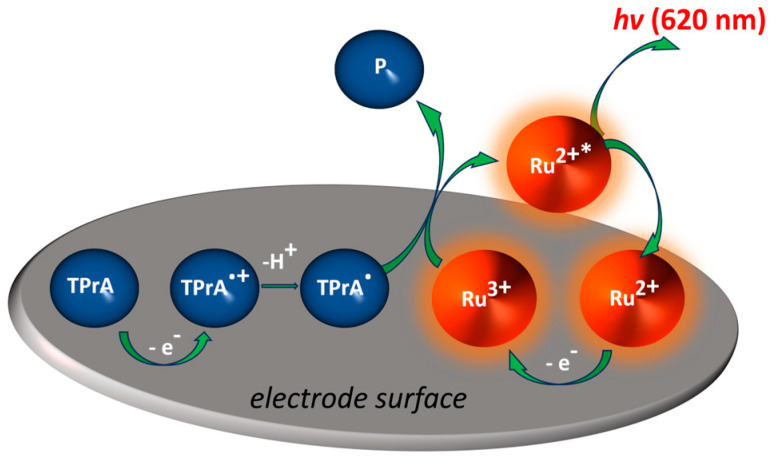
Overview of the ECL process for the most common system used, consisting of the luminophore, Ru(bpy)_3_^2+^, and the tri-n-propylamine (TPrA) co-reactant. The ECL reagents are generated in situ at the electrode using cyclic voltammetry or chronoamperometry by sweeping the applied potential. Upon the oxidation of both the luminophore and co-reactant, the formed activated species further interact to form the excited state Ru(bpy)_3_^2+∗^ resulting in an ECL emission. Ru^2+^ represents Ru(bpy)_3_^2+^.

Current (bio)sensors for the detection of pollutants aim to replace classical detection techniques based on liquid and gas chromatography or mass spectrometry by providing the coupling of the accuracy of measurements equivalent to such instrumental methods with the portability, affordability, and simplicity of analysis. As already mentioned, ECL is an electrochemical process in which an electron-transfer reaction taking place at an electrode surface triggers light emission. In co-reactant ECL, the emission of light is generated via a charge transfer between the electrochemical reaction intermediates of both the emitter and co-reactant (Figure 1). 

The most widely used system applied for analytical purposes is composed of the luminophore species Ru(bpy)_3_^2+^, or its derivative, and TPrA as a co-reactant. An ECL co-reactant can be defined as a reagent that, following oxidation or reduction, is able to decompose, producing highly reactive reductive or oxidative species (Figure 1), which can proceed through an electron transfer reaction with an oxidized or reduced luminophore in order to generate ECL. Combining electrochemistry and luminescence in this smart way enables the unique advantages of ECL compared to other optical sensing methods, as a light source is not necessary, and this makes the detection set-up simpler and most importantly, no background signal is generated from scattered light, and luminescent impurities. As ECL does not require a light source, it simplifies the detection apparatus, and, most importantly, invalidates background signals from scattered light and luminescent impurities, thus providing improved sensitivity. However, some specific ECL configurations employing semiconductors and named photo-induced ECL require an excitation light to photo-generate holes and electrons that trigger the emission of ECL [48,49,50]. These properties have resulted in ECL becoming a significant detection method in analytical chemistry and microscopy [23,30,51,52,53,54,55,56,57,58,59,60]. Commercial ECL systems for clinical diagnostics using standard ECL pair, Ru(bpy)_3_^2+^, or one of its derivatives, and TPrA run over 1.3 billion tests per year [26,60].

Organometallic compounds are in focus for the development of ECL-emitting species due to their ECL nature. In order to advance the sensitivity, stability, and reproducibility of ECL biosensors, it is particularly important to optimize luminophores in terms of robust ECL signals and stability. Conventional luminophores, including luminol, Ru(bpy)_3_^2+^, g-C_3_N_4_, and derivatives, all have excellent ECL responses, but their utilization in an ECL sensor can be affected by their stability in water solutions, or reduced contact probability with the co-reactant. Ru(bpy)_3_^2+^ species and their derivatives are considered to be the most efficient luminophores as they possess excellent electrochemical and spectroscopic properties. Ru(bpy)_3_^2+^ can be applied in both water and organic solutions depending on the counter-ion, and also immobilized on the electrode surface. For instance, Ru(bpy)_3_^2+^ is stable in the solution, and electrochemical oxidation can generate the reactive species Ru(bpy)_3_^2+^ (Figure 1). Classical anodic co-reactants are tertiary, secondary, and primary alkyl amine groups (especially TPrA or DBAE) and oxalate [29,51,52,53]. For instance, after its heterogeneous oxidation at the electrode surface or homogeneous oxidation via Ru(bpy)_3_^2+^, the TPrA^●+^ cation radical deprotonates rapidly to form the reducing neutral radical, TPrA^●^. Ru(bpy)_3_^3+^ is then reduced exergonically via TPrA^●^, forming the excited state Ru(bpy)_3_^2+∗^ (Equation (1)), which decays to the ground state and emits orange-red light [54,55,56,57].

$$\begin{array}{c} {\mathrm{Ru}(\mathrm{bpy})}_{3}^{3+}{+\mathrm{TPrA}}^{●}\\ {\mathrm{Ru}(\mathrm{bpy})}_{3}^{+}{+\mathrm{TPrA}}^{●+} \end{array}\}\to {\mathrm{Ru}(\mathrm{bpy})}_{3}^{2+*}+\mathrm{products}\begin{array}{c} \left(1\right)\\ \left(2\right) \end{array}$$

(3) $${Ru(bpy)}_{3}^{+}+{Ru(bpy)}_{3}^{3+}\;\to \;{Ru(bpy)}_{3}^{2+*}+{Ru(bpy)}_{3}^{2+}$$

Furthermore, the excited state of ${Ru(bpy)}_{3}^{2+}$ can be produced via three different routes: (1) Ru(bpy)_3_^3+^ with reduction by TPrA^●^ as explained above, given in Equation (1); (2) Ru(bpy)_3_^+^ with oxidation by TPrA^●+^ radical cation (Equations (2) and (3)); and (3) the Ru(bpy)_3_^3+^ and Ru(bpy)_3_^+^ annihilation reaction (Equation (3)).

Thus, in ECL reactions, light emissions lead to the regeneration of ruthenium complexes, making the ECL methodology reusable and highly attractive from an analytical point of view. Therefore, they act as labels for ECL bioassays. In addition, several advantages exist for immobilizing luminophores, and they include improved sensitivity due to the concentration of emitter centers in the detection region near the electrode’s surface and reduced chemical consumption, particularly impacting flow systems. Hence, the integration of luminophores in materials with a high porosity and specific surface area, such as MOF, can significantly increase the ECL sensor’s analytical performance. Even if TPrA is an effective co-reactant for Ru(bpy)_3_^2+^ ECL, there are well-known backdowns associated with its properties. Crucially, TPrA is highly toxic (LD_50_ oral: 98 mg/kg, LC_50_ inhalation: 1500 mg/m^3^) and very volatile. The co-reactant ECL technology is an essential part of all commercially available ECL analytical instrumentations. Co-reactants are more comfortable to work with not only in aqueous media but also in physiological conditions (pH~7.4). Finding new co-reactants with a high ECL efficiency for bioassays is a constant driving force in this area [25,51,58,59,60,61]. Among others, persulfate is the first example of a co-reactant ECL system produced by applying cathodic potential [62]. The application of MOFs as luminophore carriers for pollutant detection enabled the use of persulfate as a less toxic and efficacious co-reactant, reaching extremely low LOD in water pollutant detection such as femtomolar, and making the whole system more environmentally friendly [63]. Nevertheless, ECL MOFs are, therefore, promising materials for the development of clinical diagnostic assays using a non-toxic ECL system. 

## 3. MOFs for ECL Sensors

**Figure 2:**
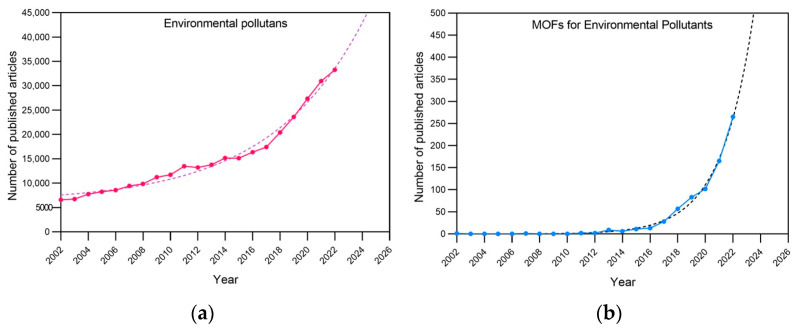
Environmental pollutants and metal–organic frameworks in the literature in the period 2002–2022. (**a**) Values were obtained by searching “Environmental pollutants” and (**b**) “Environmental pollutants-Metal–organic frameworks” in Scopus (solid lines). Trends were obtained via fitting a tendency curve and projecting it for the next 4 years (dotted lines).

Metal–organic frameworks represent an attractive group of highly ordered crystalline coordination polymers shaped via the coordination of metal ions/clusters and organic bridging linkers/ligands. Taking into account the unique structures and properties of MOFs, which include high surface area, tailorable pore size, the high density of active sites, and high catalytic activity, different MOF-based sensing platforms have been designed for environmental contaminant detection and purification involving anions, heavy metal ions, organic compounds, and gases. Figure 2 shows the articles published for “Environmental pollutant” and “MOF for Environmental Pollutant”, along with the future trends according to their publication rate in the last twenty years. 

**Figure 3:**
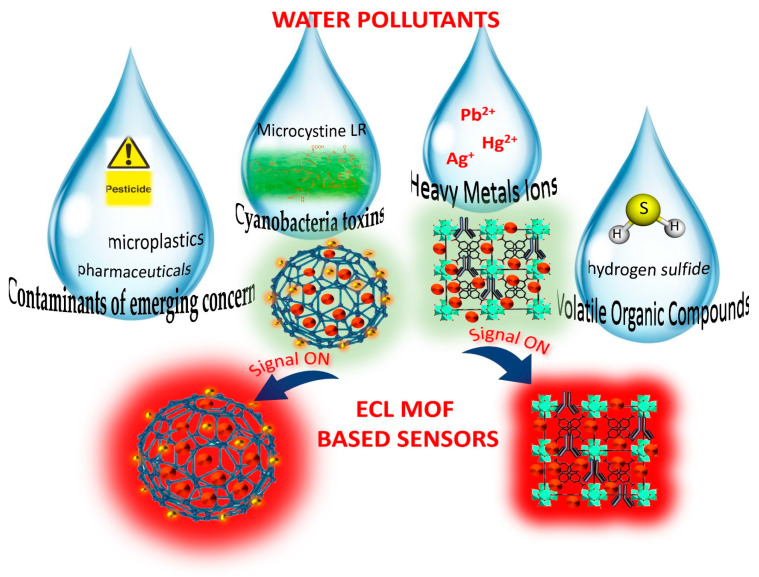
Overview of MOF structures and their applications for water pollutant assessment.

Due to their high chemical stability, MOFs have been exploited not only as a promising sensing material but also as superior adsorbents of different environmental pollutants from both soil and water. In comparison with MOFs, different porous sorbents like zeolites, activated carbon, and others have several disadvantages, including material stability, high density, a lack of structural tenability, and low uptake capacity or selectivity. MOFs of different sizes and morphologies can be controllably produced using various synthesis methods such as sonication, electrochemical, hydro/solvothermal, mechanochemical, microwave, etc., [64,65,66,67]. The diverse MOFs obtained, which can be a class of 2D or 3D microporous materials, have emerged as prominent materials for water contaminant research. In these materials, porous structures are assembled using metal cation salts or clusters linked with polydentate organic ligands with coordination-type connections. They can also be combined with other materials, such as nanoparticles, to form advanced nanocomposite materials. For instance, MOFs combined with conductive nanoparticles show exceptional electron conductivity, while MOFs alone have poor conductivity. In this review, we explore the relationship between the characteristics of ECL-active MOFs and their application for the detection of water pollutants (Figure 3). 

Electroactive luminophores can be easily incorporated in MOFs due to their nano-scale and ordered porosity or through metal ion chelation to generate ECL-active MOFs [68]. Luminophores can be incorporated in MOFs during their synthesis or via post-synthesis modifications. The integration of MOFs is achieved through functional nanomaterial design by monitoring and tuning photophysical and photochemical properties and changing the structure of organic linkers, metal clusters, and guest species. For instance, when Ru complexes are integrated into 2D MOF nanosheets during the synthesis, a significant improvement in ECL luminescence efficiency was obtained [69]. In comparison to Ru complexes alone, the resulting emitter within the composite has a high level of mobility inside frameworks with restricted intramolecular rotation and exhibits enhanced charge delocalization. Moreover, an additional increase in ECL efficiency can be obtained by integrating Ru complexes in MOFs doped with other transition metals. The doping of MOFs with transition metals improves their electrical conductivity. Zhao and coworkers [70] showed that introducing a ruthenium pyridine complex in Ni-MOFs to produce NiRu-MOFs can lead to a significant boost in ECL efficiency compared to pure Ni-MOFs. Another approach consists of the encapsulation of the luminophore during the growth of MOF. For instance, Dong et al. [71] encapsulated Ru(bpy)_3_^2+^ within mesoporous and hollow MIL-101(Al)–NH_2_ to which the co-reactant, poly(ethylenimine), was covalently linked. The co-reactant prevented luminophore leakage and enabled a self-enhanced ECL response. Post-synthesis modifications of MOFs are possible due to well-defined pore sizes and their charge via some linkers [44,68,72,73]. 

Although ECL-based MOFs just recently opened a new horizon for highly sensitive targeted bioanalysis, the performance of each of these MOFs alone does not meet the requirements for signal amplification. Recently, luminescent MOFs, which belong to the group of multifunctional MOFs, were designed as highly crystallized ECL emitters in an aqueous medium [74]. These MOFs demonstrated exceptional performance with surface state models in both the co-reactant and annihilation of ECL in the aqueous medium. In comparison with individual elements, the framework structure of multifunctional MOFs significantly upgrades the emission of ECL. A high-stability self-enhanced ECL emission can be achieved and realized by the accumulation of MOF cation radicals via pre-reduction electrolysis. These MOFs enable a proof of concept that molecular crystalline materials can be applied as new ECL emitters.

Table 1 shows some recent examples of ECL-active MOFs applied in the sensing of potentially toxic species, the linear detection range (LDR), the type of co-reactants, and the type of the real sample’s medium. Sensor performances were in accordance with provisional guideline values for pollutant concentrations in drinking water regarding the limit of detection for the adequate analytical detection method as recommended in the drinking-water quality guidelines provided by WHO [75]. 

## 4. Applying ECL-Active MOFs in Water Pollutant Sensing

### 4.1. ECL MOF Sensors for Heavy Metals Detection 

Waterbody contamination with heavy metals is a critical issue that adversely affects humans, plants, and animals. Heavy metal pollution has been found in sediments of rivers, lakes, and other waters and is a reason for significant concern because of their enrichment, concealment, persistence, and toxicity. A large number of scientific reports deal with the improvement of systems for the detection of heavy metals compared to time-consuming classical analytical techniques, including atomic absorption spectrometry and inductively coupled plasma—optical emission spectrometry. The application of MOFs as platforms for sensing and capturing heavy metals has significantly increased due to their high surface area, tunable pore chemistry, and fast adsorption kinetics. Currently, photoluminescence is the most exploited method for detecting heavy metals with MOFs, and in this case, interactions between the pollutants and MOFs modify luminescent properties [93,94]. Among the luminescent MOFs, MOFs with luminophores that exhibit ECL signals have drawn a lot of attention as emitters for the sensitive detection of heavy metals. Shan et al. [77] reported on an ECL sensor for the highly sensitive and selective detection of Pb^2+^ based on Ru(bpy)_3_^2+^ and the encapsulated UiO66 metal–organic framework. The nanocomposite contained Ru(bpy)_3_^2+^—UiO66 MOF and –NH_2_—group functionalized silica nanoparticles (NH_2_-SiO_2_). The large surface area of NH_2_-SiO_2_ ensured an excellent platform for ECL sensing. The encapsulation of Ru(bpy)_3_^2+^ in UiO66 MOF significantly enhanced the ECL efficiency of the suggested sensor. The good linear relationship of the quenched ECL intensity within Pb^2+^ concentrations in the range from 1.0 × 10^−6^ to 1.0 × 10^2^ μM with a detection limit of 1.0 × 10^−7^ μM was obtained under optimal conditions. The oxidation-initiated reductive excitation pathway (*vide supra*) represented the ECL excitation route of Ru(bpy)_3_^2+^. The co-reactant used in the generation of ECL was triethylamine (TEA) dissolved in a buffer solution. The sensor operated at pH 7.5. At a pH lower than 7.5, the signal was low due to the inhibition of the co-reactant deprotonation, while an alkaline environment caused the precipitation of Pb^2+^. The potential ECL mechanism of Ru(bpy)_3_^2+^/TEA was proposed, suggesting a similar route as in the standard system of Ru(bpy)_3_^2+^/TPrA. Regarding its application, the proposed ECL sensor displayed its detection ability with an average concentration of Pb(II) of 9.99 nM in tap water. This was in accordance with the WHO guidelines, prescribing 1 µg/L as LOD via AAS and a practical quantification limit in the region of 1–10 µg/L [75]. Moreover, the sensor showed good stability since the ECL intensity was stable for 14 scan cycles. In another study, Jin et al. [95] synthesized a silver–MOF composite (Ag-MOF) with terephthalic acid. The silver ion was used as the ECL luminophore for an aptamer sensor to detect mercury ions in water. To improve the ECL stability of the Ag-MOF, chitosan and gold nanoparticles (Au NPs) were additionally attached to the composite. The ECL response was obtained using K_2_S_2_O_8_ as a co-reactant.

**Figure 4:**
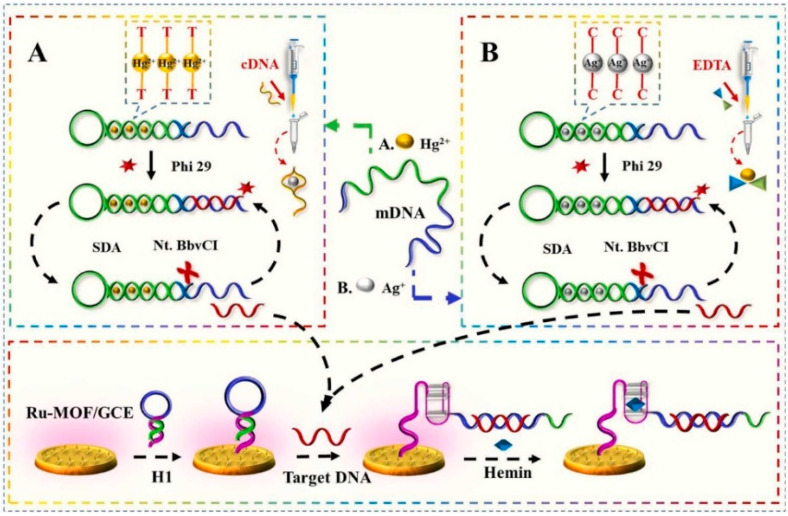
Schematic diagram of ECL ultrasensitive sensing of Hg^2+^ (**A**) and Ag^+^ (**B**). Adapted with permission from [76].

Recently, a di-functional ECL sensor utilizing Ru-MOFs and the strand-displacement-amplification reaction was proposed for the ultrasensitive detection of two heavy metal ions, Hg^2+^ and Ag^+^, using K_2_S_2_O_8_ as a co-reactant [76]. Several improvements were proposed. First, the electrochemical method was applied using Ru(bpy)_3_^2+^ and 1,3,5-benzentriic acid to prepare Ru(bpy)_3_^2+^-functionalized MOFs (Ru-MOFs) under mild conditions. In most developments, the processes applied to synthesize functionalized MOF take a long time as they include complex reaction steps and harsh conditions. Secondly, the detection step included dissolved oxygen in the reaction system and utilized its significant quenching effect on the ECL signal generated by Ru(bpy)_3_^2+^ [96]. For this, a low concentration of hemin was used as a quencher O_2_ [97,98] in order to ultimately and indirectly enhance the ECL signal. Finally, carboxyl groups in Ru-MOFs films formed on a glassy carbon electrode (GCE) were activated using EDC/NHS to immobilize the DNA H1 oligomer in the solution containing hemin. Hemin was bound to the guanine-rich part of DNA H1, forming a G-quadruplex structure (Figure 4). The ECL signal of Ru(bpy)_3_^2+^ for trace amounts of Ag^+^ was recorded after adding EDTA, while Hg^2+^ was detected in the solution containing cytosine-rich cDNA to mask Ag^+^. This ECL sensor operated at pH 7.4 and at 37 °C showed the relative standard deviation (2.62–3.37%) and recovery rates (93.43–105.49%) when applied in seawater. These values were in acceptable ranges regarding the standard regulative for these two heavy metals. The sensor had reliable storage stability when placed at 4 °C and provided an unchanged signal for 30 cycles of ECL responses. 

### 4.2. ECL MOF Sensors for CEC Detection 

Contaminants of emerging concern (CEC) is a definition for different substances present in the environment that were not detected before, or at least not in significant amounts [99]. Nowadays, various CECs, including personal care products, pharmaceuticals, hormones, and industrial chemicals released into the environment. Their potential toxicity points out that additional detection methods and new regulations are needed to enable their control and prevention. Besides presenting risks to human health, the release of various CECs in nature, even at trace level concentrations, has negative effects on animals and the environment. For instance, steroid hormones can induce feminization or masculinization in aquatic fauna, while traces of antibiotics contribute to bacteria becoming more resistant. In the context that most CECs have unknown toxicity, the United States Environmental Protection Agency and the European Commission have instigated EDSP and REACH programs, respectively, to investigate the toxicity and endocrine disruptive properties of different CECs. By contrast, many developing and underdeveloped countries still do not have such programs despite the increasing presence of pharmaceuticals, chemicals, and hormones in domestic sewage and surface water sources. 

**Figure 5:**
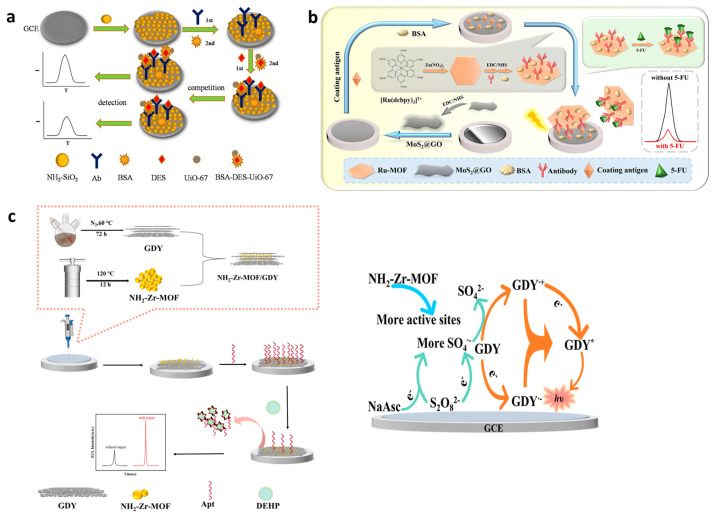
ECL MOFs for CEC detection. (**a**) The ECL sensor fabrication and sensing mechanism for DES detection. Adapted with permission from [86]. (**b**) The ECL immunosensor fabrication process and sensing mechanism for 5-FU detection. Adapted with permission from [90]. (**c**) The DEHP ELC sensor fabrication steps (**left**) and schematic diagram of the ECL signaling mechanism (**right**). Adapted with permission from [89].

The quantitative analysis of CECs requires sophisticated and sensitive analytical instruments, as mentioned above. MOF ECL sensors could be the answer to such limitations. In most of them, recognizing the need to target CEC relies on the utilization of specific antibodies or aptamers that are immobilized in MOFs. For example, a new competitive ECL immunosensor platform has been designed for the detection of diethylstilbestrol (DES) via the encapsulation of Ru(bpy)_3_^2+^ in UiO-67 MOF [86]. The interaction with DES resulted in the activation of Ru-UiO-67 MOF as a luminophore and enhanced ECL signal emissions. DES is a synthetic non-steroidal estrogen causing reduced fertility upon in utero exposure. An electrode surface modified by amino-functionalized silica (NH_2_-SiO_2_) was coated using the antibody DES and served as an immunosensing platform. In the constructed immunosensor, DES was contested with bovine serum albumin-diethylstilbestrol (BSA-DES) for binding to antibody-specific sites (Figure 5a). An increase in the unlabeled DES antigen concentration resulted in a decrease in the number of available paratopes for the Ru-MOF-labeled antigen and, therefore, the generated ECL signal. The operating conditions comprised the following: pH 7.5, 10 mM tripropylamine co-reactor, 15 mg/mL BSA/DES/Ru(bpy)_3_^2+^/UiO-67, and 2 h incubation time. The novel fabricated immunosensor is a good potential candidate for the detection of other types of biological hormones with a proven wide linear range from 0.01 pg/mL to 50 ng/mL and an LOD of 3.27 fg/mL.

Promising 2D ruthenium-MOF nanosheets were synthesized for the ultrasensitive detection of fluoropyrimidine 5-fluorouracil (5-FU) in an ECL competitive-type immunosensor [90]. The 5-FU is an anticancer drug and is particularly and widely applied for the treatment of colorectal cancer. A larger surface area of 2D Ru-MOF nanosheets led to the increased loading of Ru(dcbpy)_3_^2+^, exposing more bindable active sites and thus improving the performance of MOFs as ECL emitters. Electrode surface modification with thin 2D molybustion/graphene oxide (MoS_2_@GO) improved the electron transfer rate of the electrode used as the sensing platform due to the MoS_2_ graphene-like structure (Figure 5b). The large specific surface area of MoS_2_@GO and its piezoelectric catalytic efficiency further provided the loading of more 5-FU coating antigens. The competition between free 5-FU- and 5-FU-coating antigens on the sensor platform for the binding sites in Ru-MOF/antibodies was the competitive immunoassay strategy. The ECL signal was efficiently generated via a cathodic ECL route using persulfate as a co-reactant with the 0.4 mg/mL MoS_2_@GO and 12 µg/mL 5-FU antibody and 8 µg/mL 5-FU antigen as the operating conditions. The proposed immunosensor showed a high sensitivity, wide detection range (0.0001 ng/mL–100 ng/mL), and low limit of detection of 0.031 pg/mL. The sensing platform could be adapted for the detection of other types of drugs if other specific antibodies were used. The sensor exhibited excellent stability under five consecutive scans. 

Another example includes an ECL aptamer sensor with NH_2_-Zr-MOF for ultra-sensitive detection of a plastic additive—di-(2-ethylhexyl)phthalate (DEHP) [89]. Merging a highly efficient electrocatalytic NH_2_-Zr-MOF and graphdiyne (GDY) composite onto a glassy carbon electrode surface notably enhanced the complete electrochemically active surface area and consequently improved ECL’s signal intensity. Lamellar GDY was used as the luminescent body, combined with NH_2_-Zr-MOF to modify the surface of a GCE, followed by the fixing of the carboxylated aptamer (COOH-Apt) that could specifically identify the target on NH_2_-Zr-MOF via the amide bond (Figure 5c, left panel). Sodium ascorbate (NaAsc) was added as a co-reactant accelerator for the GDY/S_2_O_8_^2−^ system (Figure 5c, right panel). The analytical functionality of the sensor to recognize DEHP was obtained with 16 µM of NaAsc, 3µM of aptamer immobilized onto the electrode, and 20 min of incubation time. The ultra-sensitive detection of DEHP in the linear range of 1.0 × 10^−12^ to 1.0 × 10^−4^ mg/mL with an LOD of 2.43 × 10^−13^ mg/mL of DEPH was achieved. The practical application of the sensor was shown in river water and urban drinking water samples in which the presence of the DEPH pollutant raises potential health hazards.

Li and coworkers [100] developed an ultrasensitive and selective method for detecting bisphenol A (BPA) using a solid-state ECL aptasensor. This aptasensor utilized titanium-based MOFs, namely MIL-125, as a carrier for the luminescent Ru(bpy)_3_^2+^. Ru(bpy)_3_^2+^ encapsulating MIL-125 (Ru(bpy)_3_^2+^@MIL-125), which was applied onto a glassy carbon electrode and functioned as the working electrode. The best operating conditions were obtained when the electrode was modified with 2.5 µL of Ru(bpy)_3_^2+^@MIL-125. To enhance selectivity, a thiol-based aptamer specific to BPA was attached to the working electrode through a Ti-S bond. The specific binding between the aptamer and BPA resulted in the significant quenching of the ECL signal. This led to the development of a selective ECL aptasensor for BPA. The ECL aptasensor utilizing Ru(bpy)_3_^2+^@MIL-125 demonstrated a strong ECL response for detecting BPA. Under optimal conditions, the aptasensor displayed a wide linear detection range from 1.0 × 10^−12^ to 1.0 × 10^−6^ M, with an excellent detection limit of 6.1 × 10^−13^ M. Furthermore, the ECL aptasensor was able to selectively detect BPA even in the presence of other BPA interference compounds in a mixture. Nevertheless, the sensor had a relatively high cost because MOF synthesis was time-consuming and complicated. 

In the study by Wen et al. [83], a sensitive and selective ECL aptasensor was developed using Co-Ni/MOF to enhance the detection of chloramphenicol (CAP). Black phosphorus quantum dots (BPQDs) were synthesized and introduced into the precursor solution to create BPQDs-doped PTC-NH2 nanoparticles (BP/PTC-NH2) as ECL emitters. The Co-Ni/MOF showed a significant improvement in signal amplification compared to BP/PTC-NH_2_. Under the optimized operating conditions of 25 min of incubation time, a 100 mV/s scanning rate, and 2µM aptamer concentration, the aptasensor successfully detected CAP within a concentration range of 1.0 × 10^−13^ M to 1.0 × 10^−6^ M with a low detection limit of 2.9 × 10^−14^ M. The developed aptasensor also demonstrated the selective detection of CAP in the presence of interference compounds, showcasing its potential applications in detecting antibiotics in aquatic environments.

**Figure 6:**
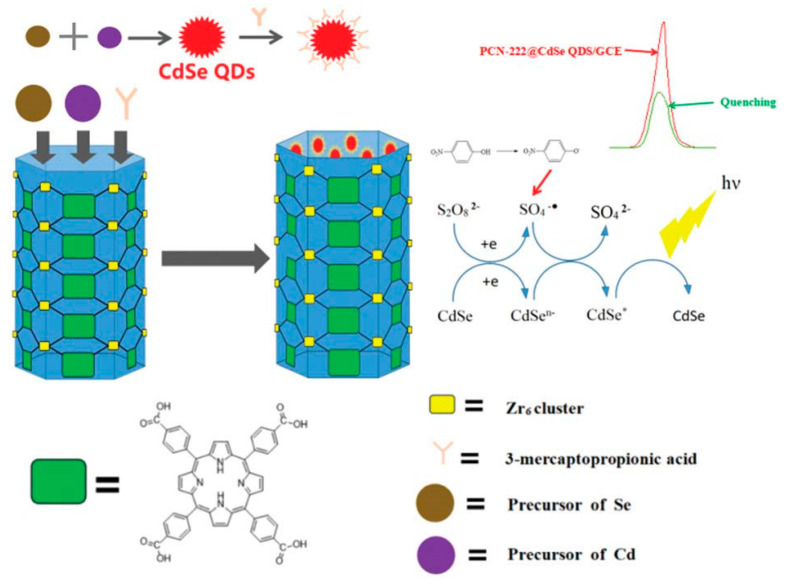
The ECL composite sensor construction process for PNP detection. Adapted with permission from [91].

Recently, an innovative method for the successful loading and anchoring of CdSe quantum dots in the pores of a Zr-based porphyrin MOF using the solvothermal method was developed for p-nitrophenol (p-NP) detection [91]. p-NP is one of the priority pollutants on the U.S. Environmental Protection Agency list because it is a carcinogen and potential endocrine disruptor, which tends to persist in water. The novel compound material is known as PCN-222@CdSe was characterized by a greatly increased ECL signal intensity and luminescence stability in comparison with single CdSe quantum dots. p-NP effectively quenched the ECL signal of PCN-222@CdSe (Figure 6). The operating conditions comprised 4 mg/mL of PCN-222@CdSe, a 100 °C reaction temperature, and a 60 min reaction time. The ECL sensor was able to sensitively and efficiently identify nitrophenol compounds in the range of 100 ppm to 0.1 ppb, with an LOD as low as 0.03 ppb. It was successfully tested for samples of tap and lake water. 

### 4.3. ECL MOF Sensors for VOC Detection 

**Figure 7:**
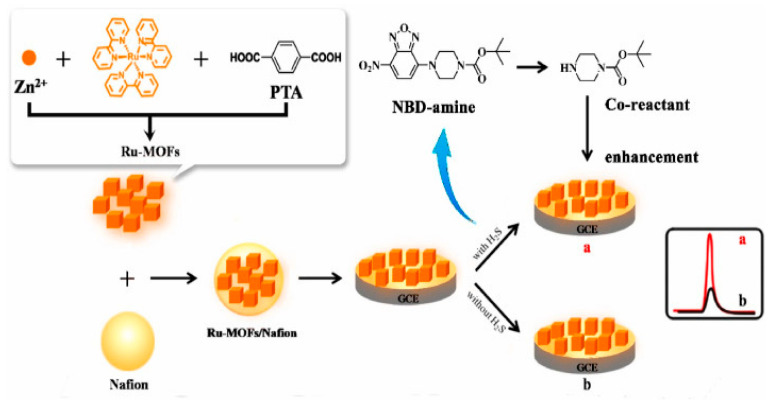
The construction and sensing mechanism of an ECL sensor for H_2_S detection. Adapted with permission from [78].

Volatile organic compounds, such as acetone, benzene, xylenes, and toluene, are primarily emitted from industrial chemical processes, vehicles, various home care products, and building/construction materials. In addition, there are some naturally occurring sources of VOCs, such as mono- and tri-bromomethane, mono- and di-chloromethane, and chloroform that are metabolically active products of marine organisms. The production of VOCs is influenced by the type of sediment [101]. Typically, sediments with more sorptive properties include muddy sediments that capture and accumulate VOCs (like dimethyl sulfide and methyl mercaptan), while less sorptive sandy sediments contain very low amounts of VOCs. Some VOCs are highly toxic when absorbed through the gastrointestinal tract or when they penetrate into the organism by crossing the skin barrier. In addition, VOCs can rapidly evaporate into the air and cause severe intoxication via inhalation. Besides causing severe harm to human health, VOCs have a negative environmental effect since they can deplete the ozone. Consecutively, highly toxic VOCs are subject to strict regulations, and their monitoring is of the utmost importance. Besides laboratory-based instruments for VOC detection and quantification, many sensors have been validated and are commercially available such as those based on photo-ionization or electrochemistry [102]. Although the utilization of MOF-based colorimetric sensors for VOC detection has been extensively studied, up until now, only a few MOF ECL sensors have been explained. Recently, the adsorption of small molecules via the porous structure of Ru-MOFs was used for the sensitive ECL detection of H_2_S [78]. Ru(bpy)_3_^2+^ was encapsulated within a multifunctional MOF, together with novel co-reactants in the form of NBD-amine, as a recognition probe. In the presence of H_2_S, NBD amine released the secondary amine and enhanced the ECL signal of Ru-MOFs immobilized onto GCE (Figure 7). The increased ECL signal was proportional to the concentration of H_2_S. In this sensor, MOFs as nanocarriers efficiently increased the load and amount of Ru(bpy)_3_^2+^, whose large specific surface area could adsorb more H_2_S on the GCE surface and, thus, produce a greater amount of secondary amine as co-reactants. The encapsulation of Ru(bpy)_3_^2+^ improved its interaction with the co-reactant, contributing to the enhancement of the ECL signal. As a result, the proposed ECL sensor detected H_2_S with the dynamic range from 10^−11^ M to 10^−4^ M and an LOD of 2.5 × 10^−12^ M at 180 min of the reaction time. Moreover, the sensor was stable under continuous cyclic potential scans for 10 cycles. 

### 4.4. ECL MOF Sensors for Cyanotoxin Detection in Water

**Figure 8:**
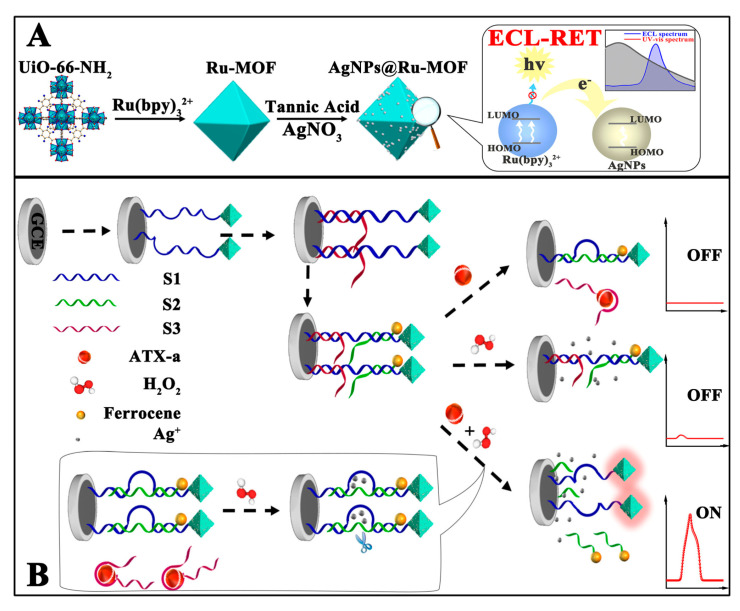
Schematic presentation of in situ generation of AgNPs@Ru-MOF and progress of ECL-RET (**A**) and the response process of the ECL-RET aptasensor for anatoxin-a (**B**) Adapted with permission from [79].

The rapid proliferation of cyanobacteria in weakly circulating water regularly leads to water contamination with cyanotoxins that can be classified as hepatotoxines, dermatotoxicines, hepatotoxins, and neurotoxicines. Anatoxins and saxitoxins are the most important neurotoxins [103,104]. Anatoxin-a is an alkaloid produced from a variety of cyanobacteria, provoking seriously harmful effects that can be fatal. Although currently there is no set low value for the allowed anatoxin-a content in drinking water, its accurate detection enables the efficient control of water quality and prevention of poisoning. Xia et al. [79] reported a DNAzyme-based, target-triggered, redox-controlled responsive ECL resonance energy transfer (RET) aptasensor for anatoxin-a detection. The system utilized Ru(bpy)_3_^2+^-doped MOF as an energy donor and AgNPs/ferrocene as dual-energy acceptors. A Ru(bpy)_3_^2+^ chromophore was encapsulated into zirconium(IV)-based MOFs, followed by the coating of AgNPs as a primary ECL-RET quencher. Three DNA strands were considered as follows: the first was a thiolated DNAzyme strand that linked the formed AgNPs@Ru-MOF, the second was an oligomer modified with ferrocene, and the third was an anatoxin-a specific aptamer that served as a secondary quencher. When AgNPs@Ru-MOF and three DNA strands were assembled on the electrode, the recorded ECL background was extremely low due to dual quenching. However, the binding of anatoxin-a increased the ECL signal intensity significantly by exposing the Ag^+^ ion and simultaneously moving away ferrocene from Ru-MOFs (Figure 8), which restored ruthenium luminescence. The sensor was operative directly in lake and river waters and was stable under continuous scanning for 15 cycles. 

Microcystins produced by cyanobacterial bloom are heptapeptide toxins that are distributed most widely and pose a severe threat to the quality of drinking water. Microcystins are a type of monocyclic heptapeptide and possess many isomers that are attributed to different compositions of the two variable amino acids in polypeptides. Among them, MC-LR exhibits the strongest toxicity [105]. A highly sensitive ECL-based apta-sensor was developed to detect MC-LR in water [80]. The on–off–on signal strategy relied on Ru-Cu MOF as the ECL signal-transmitting probe and three types of PdPt alloy core–shell nanocrystals as signal-off probes. Bimetallic-hybridized MOFs (RuCu MOFs) were synthesized as an ECL signal emitter.

**Figure 9:**
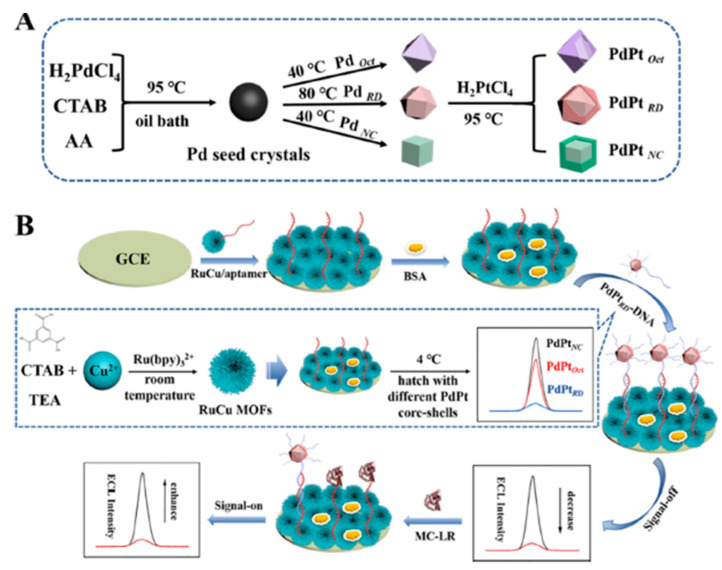
Left part: synthesis process of different core–shell NPs (**A**) Illustration of the on–off–on ECL sensing model. (**B**) Right part: the HRTEM image (**A**,**B**) of Cu-MOFs, the SEM. Adapted with permission from [80].

The high porosity combined with intrinsic crystallinity was achieved by combining the ruthenium bipyridyl with the copper-based MOF (Cu-MOF) precursor. Since bipyridine ruthenium in RuCuMOFs has the ability to perform energy transfer to the organic ligand (H3BTC), this resulted in an ultra-efficient ligand luminescent ECL signal probe, which exceptionally improved the aptasensor’s sensitivity. The quenching effects of noble metal nanoalloy particles with different crystal states obtained using a seed-mediated growth method further improved the sensitivity of the aptasensor—PdPt octahedral (PdPtOct), PdPt rhombic dodecahedral (PdPtRD), and PdPt nanocubic (PdPtNC) nanoparticles (Figure 9A). Among them, the PdPtRD nanocrystal showed higher activity when combined with excellent durability as a result of the charge redistribution due to the hybridization of Pt and Pd atoms. 

Furthermore, PdPtRD exposed more active sites with a large specific surface area, resulting in the higher loading of –NH_2_–DNA strands. Outstanding sensitivity and stability in MC-LR detection were shown via the fabricated aptasensor, including a linear detection range of 0.0001–50 ng/mL (Figure 9B). It was applied in tap water under operating conditions of pH 7.4, a low concentration of the toxic co-reactant TPrA of 7 mM, and only 0.25 mg/mL of RuCu MOFs. The application of alloy nanoparticles of noble metals and bimetallic MOFs opens new perspectives in the field of ECL immunoassays.

## 5. Conclusions

In this review, we present some developments in ECL sensors using MOF luminophores for water quality assessment. ECL-active MOFs have been developed progressively, and the majority of the results presented here have been published recently. Porous MOFs have varied functional groups and a large specific surface area, providing a variety of modification strategies for loading ECL molecules. Taking into account the fact that waterbodies may be contaminated with toxic pollutants in trace-level concentrations, the main requirement of ECL-active MOFs is high sensitivity. Advanced analytical ECL performances can be achieved via the incorporation of luminophores and nanoparticles as functional moieties in multifunctional MOFs. In addition, specificity and selectivity can be further promoted via signal amplification and/or on–off quenching strategies. Such ECL-sensors based on multifunctional and multicomponent MOFs benefit the versatile MOF characteristics, providing the possibility of fine-tuning donor and acceptor distances in the sensor with a key impact in determining the ECL analytical performances. In addition, MOFs provide good channels for the transport of co-reactants, electrons, and ions. Indeed, the integration of luminophores into frameworks results in the confinement-enhancement of the luminescence signal properties due to the distribution of molecules and intramolecular energy transfer. Moreover, the electrochemical activation of luminophore is facilitated in configurations where the luminophore is directly connected to metal within MOFs, leading to low potential ECL emissions. 

In most cases, the recognition of water pollutants relies on a specific antibody or aptamer associated with MOFs. This is a limiting feature for the future development of ECL-active MOFs, as only restrictive numbers of such molecules are available. In addition, being biological molecules, both aptamers and antibodies need to fold into a specific conformation to be active. Variations in the pH, temperature, or salt content may unfold and inactivate them. This has to be taken into account when a specific aptamer or antibody is associated with MOFs. Although many studies have shown the high selectivity and detection of ECL-active MOFs, their refined selectivity on chemically similar molecules is rarely tested. The future development of MOFs with differentiated affinities towards different toxic species allows combinations of multiple ECL-active MOFs in a sensor array for more complete water analysis. Finally, sustainable applications of ECL-active MOFs have to consider sensor reusability over the efficiency of detection. 

## Figures and Tables

**Table 1:** MOF-based ECL sensing of water pollutants.

MOF Type	Analyte	Limit of Detection (LOD)	Type of MOF Synthesis	Linear Detection Range (LDR)	Co-Reactants	Medium	Reference
Ru-Zn: MOF Ru(bpy)_3_^2+^ 1,3,5-benzentriic acid	Ag^+^/Hg^2+^	0.00298–0.00032 pM	Electrodeposition, electrochemical synthesis	0.001–1000 pM/ 0.01–10,000 pM	K_2_S_2_O_8_	Seawater, water	[76]
Ag-MOF@CS @(Au-NPs)	Hg^2+^	66 fM	Ultrasonic, solvothermal	300 fM–1 μM	K_2_S_2_O_8_	Water, lake water	[63]
NH_2_-SiO_2_/Ru(bpy)_3_^2+^-UiO66	Pb^2+^	1.0 × 10^−7^ μM		1.0 × 10^−6^– 1.0 × 10^−2^ μM	TEA	Water, tap water	[77]
Ru-MOFs	H_2_S	2.5 × 10^−12^ mol L^−1^		1.0 × 10^−11^mol L^−1^– 1.0 × 10^−4^ mol L^−1^	NBD-amine 7-nitro-1,2,3-benzoxadiazole amine	Water, human serum samples	[78]
S2-Fc/S3/S1-AgNPs @Ru-MOF	Anatoxin-a	0.034 µg/mL	Solvothermal	0.001–1 mg/mL	TPrA	Lake and river water	[79]
Ru-Cu MOF	Microcystin-LR	0.143 pg/mL	Ultasonication	0.0001–50 ng/mL	TPrA	Tap water	[80]
Hf-MOF/Ir2PD/APS/ ITO	Acetamiprid	0.0025 nM	Directional self-assembling	0.01–10 nM	TPrA	Pakchoi	[81]
CdTe@ZnNi-MOF	Chlorpyrifos	6.23 × 10^−17^ M	Blending	1.0 × 10^−14^– 1.0 × 10^−9^ M	Luminol-O_2_	Vegetables	[82]
Co-Ni/MOF	Chloramphenicol	2.9 × 10^−14^ M	Solovothermal	1.0 × 10^−13^– 1.0 × 10^−6^ M	BP/PTC-NH_2_)/S_2_O_8_with K_2_S_2_O_8_	Tap water	[83]
Hollow Cu/Co-MOF	Acetamiprid and malathion	0.015 pM/ 0.018 pM	In situ, solvothermal	0.1 μM–0.1 pM	Luminol H_2_O_2_, K_2_S_2_O_8_	Apple and tomato	[84]
UCNPs/Pt@MOF	Diethylstilbestrol	3.8 fg/mL	Layer-by-layer growth method	0.1 pg/mL to 30 ng/mL	CBS H_2_O_2_	Tap and river water	[85]
Ru(bpy)_3_^2+^/UiO-67	Diethylstilbestrol	3.27 fg/mL	Solvothermal	0.01 pg/mL to 50 ng/mL	TPrA	Urine	[86]
Eu(II)-MOFs	Trenbolone	4.42 fg/mL		10 fg/mL–100 ng/mL	TPrA	River water	[87]
CDs@HKUST-1	Catechol	3.8 × 10^−9^ mol/L	Hydrothermal synthesis	5.0 × 10^−9^–2.5 × 10^−5^ mol/L	K_2_S_2_O_8_	Tea sample	[88]
NH_2_-Zr-MOF	DEHP	2.43 × 10^−13^ mg/mL		1.0 × 10^−12^– 1.0 × 10^−4^ mg/mL	K_2_S_2_O_8_	River and urban drinking water	[89]
Ru-MOF	5-fluorouracil	0.031 pg/mL	Ultasonication	0.0001–100 ng/mL	K_2_S_2_O_8_	Serum	[90]
PCN-222@CdSe	p-PNP	0.03 ppb	Solvothermal	100 ppm to 0.1 ppb	K_2_S_2_O_8_	Lake and tap water	[91]
PtNPs@Ce-MOFs	Trenbolone	3.61 fg/mL	One-pot solvothermal	10 pg/mL–100 ng/mL	K_2_S_2_O_8_	River water	[92]

## Data Availability

Not applicable.
